# Allogeneic Stem Cell Transplant for Acute Myeloid Leukemia: Evolution of an Effective Strategy in India

**DOI:** 10.1200/JGO.2016.006650

**Published:** 2017-02-08

**Authors:** Abhijeet Ganapule, Sandeep Nemani, Anu Korula, Kavitha M. Lakshmi, Aby Abraham, Alok Srivastava, Poonkuzhali Balasubramanian, Biju George, Vikram Mathews

**Affiliations:** **All authors:** Christian Medical College, Vellore, India

## Abstract

**Purpose:**

There are limited data from developing countries on the role and cost-effectiveness of allogeneic stem cell transplantation (allo-SCT) for patients with acute myeloid leukemia (AML).

**Patients and Methods:**

We undertook a retrospective descriptive study of all patients with AML who underwent allo-SCT from 1994 to 2013 at our center to evaluate the clinical outcomes and cost-effectiveness of this therapeutic modality.

**Results:**

Two hundred fifty-four consecutive patients, median age 34 years, who underwent allo-SCT at our center were included in this study. There were 161 males (63.4%). The 5-year overall survival (OS) and event-free survival for the entire cohort was 40.1 ± 3.5% and 38.7 ± 3.4%, respectively. The 5-year OS for patients in first (CR1), second, and third complete remission and with disease/refractory AML was 53.1 ± 5.2%, 48.2 ± 8.3%, 31.2 ± 17.8%, and 16.0 ± 4.4%, respectively (*P* < .001). From 2007, reduced intensity conditioning (RIC) with fludarabine and melphalan (Flu/Mel) was used in a majority of patients in CR1 (n = 67). Clinical outcomes were compared with historical conventional myeloablative conditioning regimens (n = 38). Use of Flu/Mel was associated with lower treatment-related mortality at 1 year, higher incidence of chronic graft-versus-host-disease, and comparable relapse rates. The 5-year OS and event-free survival for Flu/Mel and myeloablative conditioning group was 67.2 ± 6.6% versus 38.1 ± 8.1% (*P* = .003) and 63.8 ± 6.4% versus 32.3 ± 7.9% (*P* = .002), respectively. Preliminary cost analysis suggests that in our medical cost payment system, RIC allo-SCT in CR1 was likely the most cost-effective strategy in the management of AML.

**Conclusion:**

In a resource-constrained environment, Flu/Mel RIC allo-SCT for AML CR1 is likely the most efficacious and cost-effective approach in a subset of newly diagnosed young adult patients.

## INTRODUCTION

Allogeneic stem cell transplantation (allo-SCT) is the preferred consolidation therapy in selected subsets of patients with acute myeloid leukemia (AML) who are in first complete remission (CR1).^1-3^ All patients beyond first relapse will need an allo-SCT. Clinical outcomes of allo-SCT beyond first relapse will vary depending on whether second remission (CR2) is achieved, the depth of remission, and whether a patient during the process of salvage chemotherapy develops significant comorbidities, such as a fungal infection or organ dysfunction.^2,4^ Allo-SCT possibly cures AML by both cytoreduction of the conditioning regimen and the immunologic graft-versus-leukemia (GVL) effect.^2,5^ Whereas multiple studies, including meta-analysis, suggest that allo-SCT in CR1 is the best option for consolidation in high- and intermediate-risk patients with AML,^6,7^ there is still considerable debate as to whether allo-SCT should be deferred to CR2.^3^ Deferring allo-SCT to CR2 is limited by the substantial number of patients who fail to achieve CR or who acquire comorbidities that preclude them from undergoing allo-SCT.^3,4^

Historically, myeloablative conditioning (MAC) regimens were used, but they were associated with high transplant-related mortality (TRM) and graft-versus-host-disease (GVHD).^8^ To ameliorate these adverse effects and reduce nonrelapse complications, reduced intensity conditioning (RIC) and nonmyeloablative conditioning regimens were developed that rely predominantly on GVL effect for leukemia cure.^9^ Various studies have shown noninferior outcomes with RIC compared with MAC regimens in terms of overall survival (OS) and relapse rates with favorable toxicity profile,^10^ and have generally been preferred for the elderly and in patients with comorbidities.^11^

The number of patients who undergo SCT as well as the number of SCT centers are steadily increasing in India. In our experience, performing early SCT after initial induction chemotherapy in patients with AML would be cost-effective compared with salvage chemotherapy followed by allo-SCT in the event of relapse. This is especially relevant in our country where a predominantly self-pay medical care system exists and most patients can afford only one approach at a curative therapy.^12^ However, we also have additional resource constraints, such as a limited number of beds in intensive care units as well as a high incidence of multidrug-resistant bacterial infections and fungal infections after any cytoreductive therapy.^12^ The impact of all these factors on the clinical outcome and cost-effectiveness of allo-SCT as consolidation therapy has never been systematically evaluated in India. In an attempt to address some of these issues, we undertook this retrospective analysis of patients with a diagnosis of AML who underwent allo-SCT at our center.

## PATIENTS AND METHODS

This is a retrospective study of all consecutive patients with AML who underwent allo-SCT from January 1994 to December 2013. All medical data and billing information was taken from the computerized hospital information system maintained by Christian Medical College, Vellore. Patients with acute promyelocytic leukemia and those who underwent haplo-identical SCT were excluded from this study. This study was approved by the institutional review board. Written and informed consent was obtained from all patients.

### Diagnosis

Diagnosis of AML was performed by using the French-American-British criteria^13^ and, after 2008, with the WHO criteria for classification.^14^ Risk stratification was done by karyotyping using standard published criteria.^15^

### Remission Assessment

Remission status postchemotherapy was documented on the basis of criteria laid down by Cheson et al^16^ and European LeukemiaNet.^17^ Primary induction failure was defined as patients who experienced a failure to achieve remission after two induction chemotherapies. CR1 was defined as remission achieved within two consecutive induction chemotherapy regimens. CR2 and CR3 were defined as remission after receiving salvage chemotherapy for first or second relapse, respectively. Refractory AML was defined as patients with primary induction failure and those who did not achieve remission after salvage chemotherapy.

### Conditioning Regimen

Allo-SCT was offered to intermediate- and high-risk patients in CR1 and to all patients after first relapse—after salvage chemotherapy and an attempt at achieving CR2—provided they had an HLA matched donor and the financial resources to proceed with the procedure. Allo-SCT was also offered to those who were relapsed and refractory. Conditioning regimens varied according to the status of the patient at transplant. Historically, all patients in CR1 received the MAC regimen with oral busulfan 1 mg/kg/dose in four doses per day from days −7 to −4 and cyclophosphamide 60 mg/kg intravenously on days −3 and −2 (Bu/Cy). From 2007 onwards, patients in CR1 received RIC regimen with fludarabine 30 mg/m^2^/d intravenously from days −6 to −2 and melphalan 140 mg/m^2^ intravenously on day −1 (Flu/Mel). However, for a minority of patients who had high-risk cytogenetics at diagnosis, secondary AML, and patients who required more than one induction chemotherapy course to achieve remission underwent transplantation using a reduced toxicity MAC regimen with fludarabine 40 mg/m^2^/d intravenously from days −5 to −2 and intravenous busulfan 130 mg/m^2^/d from days −5 to −2 (Flu/Bu). Risk categories used for administering Flu/Bu reduced toxicity MAC in CR1 patients were similar to the recently developed and reported high disease risk index.^18^ The conditioning regimen used for patients in CR2 was initially Bu/Cy, which was later replaced by the Flu/Bu regimen. In patients in CR3, with active disease, or with refractory disease, conditioning regimens used were heterogeneous, depending on the general condition, residual disease, and at the physician’s discretion.

### Stem Cell Source

Since 2007, all patients received a peripheral-blood stem cell graft, whereas before that, most patients received bone marrow graft. For bone marrow graft, the targeted cell dose was a total nucleated cell dose ≥ 3 × 10^8^/kg, and for a peripheral-blood stem cell graft, targeted cell dose was CD34 ≥ 6 × 10^6^/kg.

### GVHD Prophylaxis

GVHD prophylaxis regimen consisted of cyclosporine and short-course methotrexate in all patients, with the exception of those who received Flu/Bu conditioning where tacrolimus replaced cyclosporine. For patients with residual disease at the time of transplant, doses of methotrexate were reduced or omitted at the physician’s discretion.

### Supportive Care

All patients were nursed in a positive-pressure, HEPA-filtered transplantation unit. All patients were started on acyclovir, cotrimoxazole, and penicillin G in the peritransplant period as prophylaxis against herpetic, *Pneumocystis* caranii, and bacterial infections, respectively.

### Definitions

Acute GVHD was graded according to the Glucksbergs-Seattle criteria,^19^and chronic GVHD was graded according to the Seattle criteria.^20^ TRM was defined as any death within 1 year of transplant, excluding those deaths that were a result of recurrence. Mixed chimerism was defined as ≤ 95% donor cells beyond day 28 post–allo-SCT. Event-free survival (EFS) was defined as time of transplant to an event; an event was defined as relapse, rejection, or death. Overall survival (OS) was defined as time from transplant to death as a result of any cause.

### Statistical Analysis

The χ^2^ or Fisher’s exact test was used for dichotomous variables, and *t* test or Mann-Whitney U test was used to compare differences between continuous variables. Kaplan-Meier analysis was performed for estimation of probabilities of EFS and OS after transplant, and the significance was assessed by log-rank test. All survival estimates were reported ± 1 standard error. For all tests, *P* ≤ .05 was considered significant. Statistical analysis was performed using SPSS for Windows version 16.0 (SPSS, Chicago, IL).

## RESULTS

### Demographics and Baseline Characteristics

A total of 254 patients with AML, median age 34 years (age 4 to 63 years), received allo-SCT at our center during the study period. Baseline demographic data are summarized in Table 1. One hundred twenty-nine (50.8%), 40 (15.7%), 8 (3.1%), 76 (29.9%), and 1 (0.4%) patients were in CR1, CR2, CR3, refractory, and unknown remission status, respectively, before undergoing allo-SCT. The majority of transplant donors were HLA identical siblings who accounted for 215 patients (84.6%) as well as an additional four HLA identical related nonsibling donors. There were four related partially mismatched donors and matched unrelated donors either full matched or partially matched in 31 cases (12.2%). The Flu/Mel conditioning regimen was administered to 84 patients (33.1%), whereas Bu/Cy, Flu/Bu, and other regimens were administered to 71 (28.0%), 53 (20.9%), and 46 (18.1%) patients, respectively. Acute GVHD developed in 147 patients (57.9%). Grade I to II GVHD was observed in 83 patients (56.5%), and grade III to IV GVHD was observed in 64 patients (43.5%). Chronic GVHD developed in 122 (48.0%) patients with 62 (50.8%) of these cases exhibiting extensive chronic GVHD.

**Table 1:**
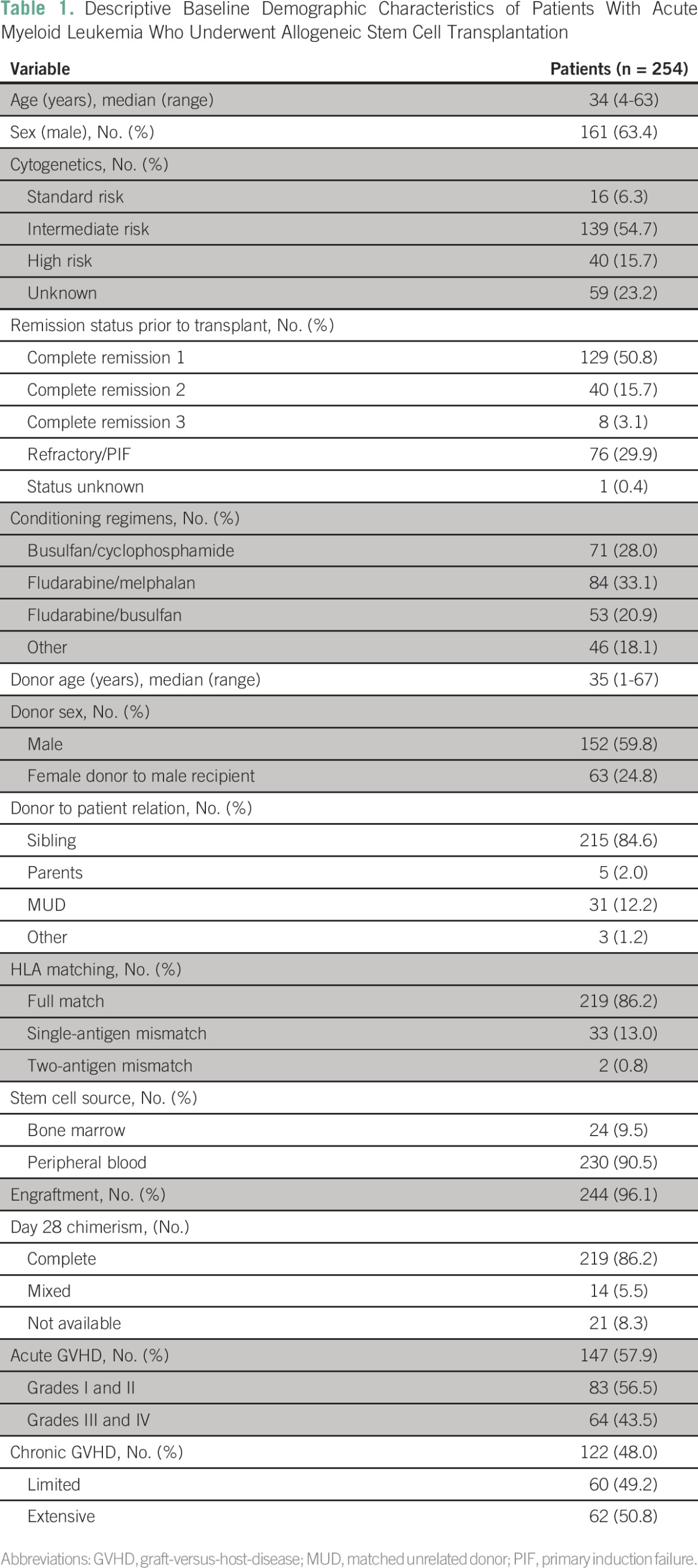
Descriptive Baseline Demographic Characteristics of Patients With Acute Myeloid Leukemia Who Underwent Allogeneic Stem Cell Transplantation

### Survival

**Fig 1:**
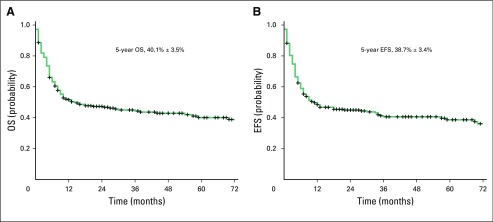
(A) Overall survival (OS) and (B) event-free survival (EFS) of the total cohort (N = 254).

**Fig 2:**
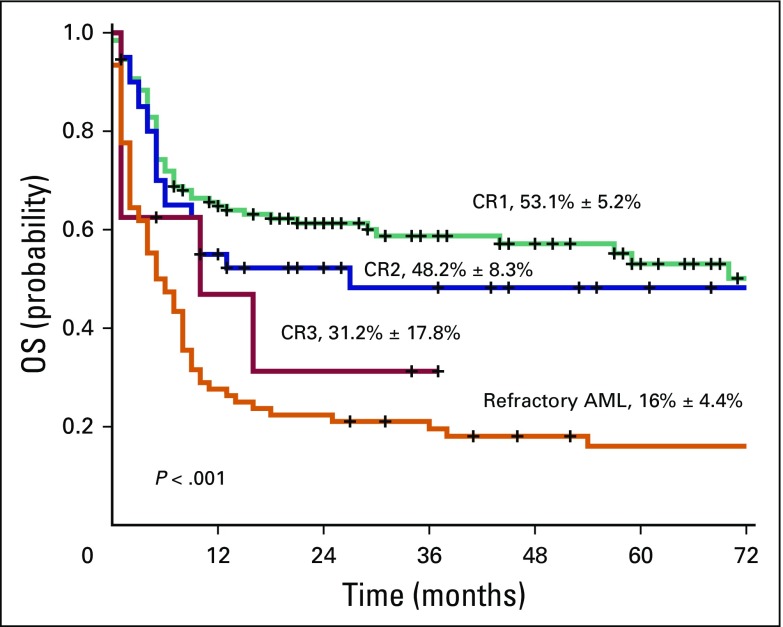
Overall survival (OS) of all patients with acute myeloid leukemia (AML) per disease status at transplantation. CR1, first complete remission; CR2, second complete remission; CR3, third complete remission.

For the entire cohort, which includes patients in CR1, CR2, and CR3 and those with refractory disease, day 100 TRM was 23.6% and 1-year TRM was 50.8%. Sixty-three patients (24.8%) experienced relapse post-transplant. The 5-year EFS and OS Kaplan-Meier estimate for the total cohort (n = 254) was 38.7 ± 3.4% and 40.1 ± 3.5%, respectively (Fig 1). The 5-year EFS and OS Kaplan-Meier estimate for the CR1, CR2, CR3, and refractory AML group was 49.0 ± 5.2%, 49.7 ± 7.9%, 33.3 ± 18.0%, and 16.5 ± 4.3% (*P* < .001) and 53.1 ± 5.2%, 48.2 ± 8.3%, 31.2 ± 17.8%, and 16.0 ± 4.4% (*P* < .001), respectively (Fig 2).

### Comparison of RIC With MAC in Patients in CR1 Allo-SCT

**Fig 3:**
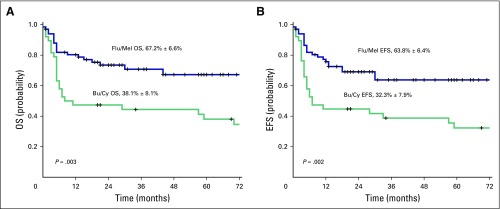
(A) Overall survival (OS) and (B) event-free survival (EFS) for patients with acute myeloid leukemia in first complete remission per conditioning regimen (fludarabine and melphalan [Flu/Mel] *v* busulfan and cyclophosphamide [Bu/Cy]).

Of 129 patients with AML in CR1, Bu/Cy, Flu/Bu, and Flu/Mel conditioning regimens were used in 38 (29.5%), 21 (16.3%), and 67 (51.9%) patients, respectively. Three patients received other conditioning regimens. To compare RIC and MAC regimens, we further compared the patients who received Flu/Mel and Bu/Cy conditioning regimens in CR1 (data summarized in Table 2). Median age of patients in the Bu/Cy group was 27 years (4 to 51 years), which was significantly younger than in the Flu/Mel group, which had a median age of 36 years (11 to 63 years; *P* = .003). All patients in the Flu/Mel group received granulocyte colony-stimulating factor mobilized peripheral-blood stem cells compared with 27 patients (71.1%) in the Bu/Cy group (*P* < .001). Acute GVHD was observed in 32 (47.8%) and 19 (50.0%) patients in the Flu/Mel and Bu/Cy arms, respectively (*P* = .842). Chronic GVHD was observed in 44 (72.1%) of 61 evaluable patients in the Flu/Mel group compared with only 11 (35.5%) of 31 in the Bu/Cy group (*P* = .001); however, of these, chronic extensive GVHD was observed in 7 (63.6%) of 11 patients in the Bu/Cy arm compared with only 16 (36.4%) of 44 patients in the Flu/Mel arm. There was a trend to a lower day 100 TRM with the Flu/Mel conditioning regimen (4 [6.0%] *v* 7 [18.4%]; *P* = .093), whereas 1-year TRM was statistically significantly lower with the Flu/Mel regimen (14 [20.9%] *v* 20 [52.6%]; *P* = .001). Relapse post–allo-SCT was not statistically significantly different between the two groups, though there was a trend to a lower risk of relapse in the Flu/Mel group (Table 2; *P* = .05). The 5-year EFS and OS Kaplan-Meier estimate for the Flu/Mel and Bu/Cy groups was 63.8 ± 6.4% versus 32.3 ± 7.9% (*P* = .002) and 67.2 ± 6.6% versus 38.1 ± 8.1% (*P* = .003), respectively (Fig 3).

**Table 2:**
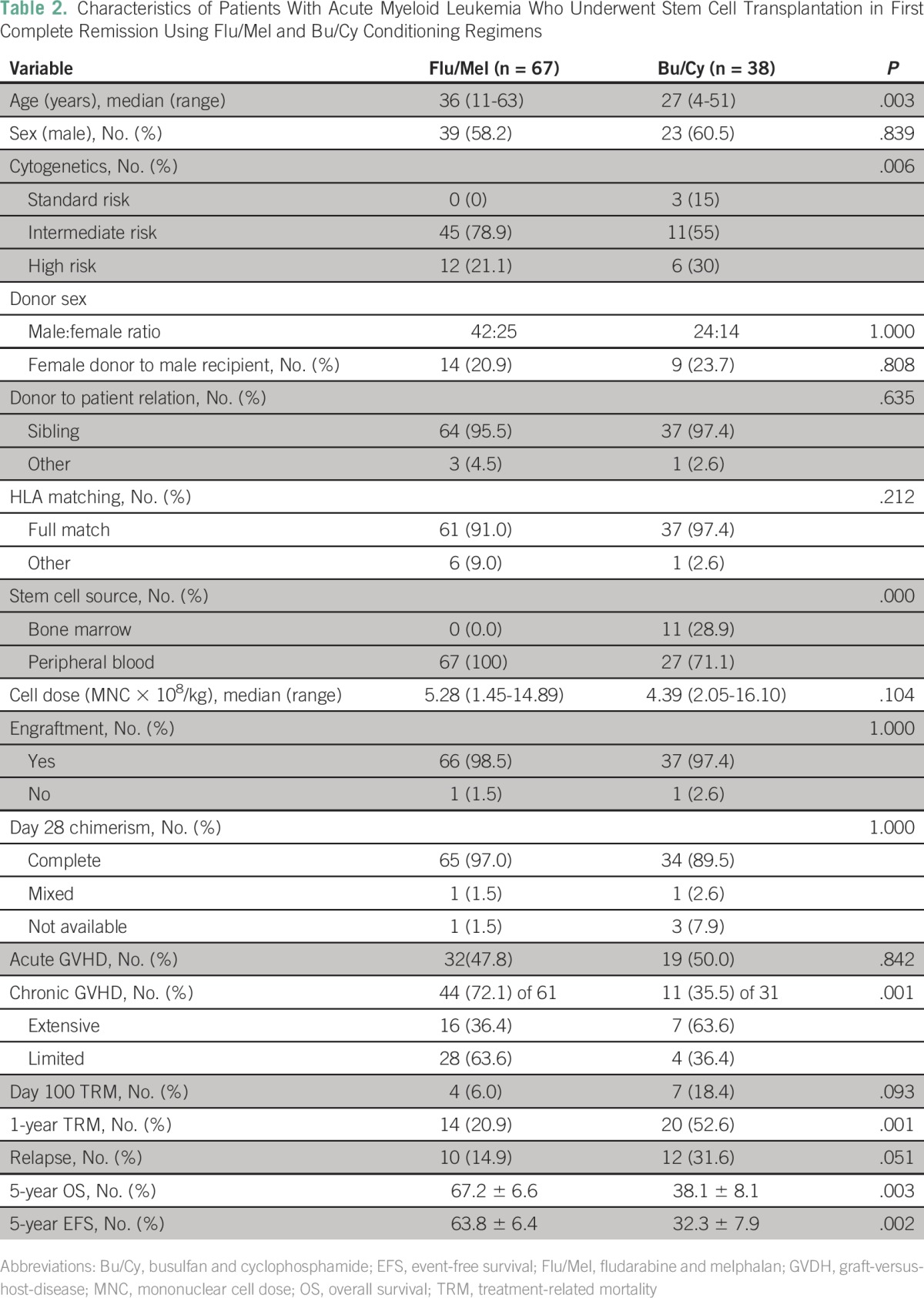
Characteristics of Patients With Acute Myeloid Leukemia Who Underwent Stem Cell Transplantation in First Complete Remission Using Flu/Mel and Bu/Cy Conditioning Regimens

### Cost Analysis of Management

For cost analysis, patients were selected randomly from the groups that received a similar consolidation therapy (chemotherapy only, n = 19; allo-SCT in CR1, n = 15) and from those that were managed post–first relapse with salvage chemotherapy followed by allo-SCT (n = 10). For comparison, only patients with matched sibling donors were included in this analysis. Total cost incurred over 1 year—from date of first contact, which includes all incurred outpatient and inpatient costs as captured comprehensively on the central hospital information system—was analyzed. Additional overhead costs, such as loss of wages, cost of relocation, and staying near the transplant center, were not available for analysis. The average cost of treatment of patients who received standard induction followed by consolidation chemotherapy alone (n = 19) and patients who underwent allo-SCT in CR1 (n = 15) was 1.52 ± 1.16 million Indian rupees (₹; $22,686 ± 17,313 USD) and 2.39 ± 1.7 million Indian ₹ ($35,671 ± 25,373 USD), respectively. Cost of treatment of patients who received salvage chemotherapy followed by allo-SCT (n = 10) was 2.53 ± 1.7 million Indian ₹ ($37,761 ± 25,373 USD), which would be the cost in addition to that incurred for initial induction and consolidation therapy before relapse ($1 USD is equal to 67 Indian ₹).

## DISCUSSION

Various studies—mainly retrospective and few prospective donor versus no donor studies—have shown the benefit of allo-SCT in patients with intermediate- and high-risk AML in CR1 but no difference in outcome in standard-risk disease compared with chemotherapy.^3,6,17,21,22^ Most of the data regarding allo-SCT in CR1 have been generated in the clinical trial setting and come from developed countries. The relevance of these observations in a developing country, such as India, with a different set of challenges has never been validated.

The median age of diagnosis of AML in our population is 40 years (range, 1 to 79 years).^12^ In the current study, 70.5% of patients were age < 40 years at the time of allo-SCT. It is well recognized that the outcome after undergoing allo-SCT is superior in young adults compared with the elderly.^3^ Deferring allo-SCT to CR2 is not ideal. In a previously reported study of patients with AML who were enrolled in MRC AML 10, AML 12, and AML 15 trials, 1,271 of 3,919 patients experienced relapse after achieving CR1 (without allo-SCT). Of these, 45% could not achieve CR2, and of 642 patients who achieved CR2, only 433 underwent allo-SCT.^4^ The counterargument against this would be the relatively high TRM with allo-SCT; however, steady improvements in supportive care and RIC regimens have steadily reduced the TRM. In a study published that compared transplant outcomes in patients with allo-SCT from 1993 to 1997 and 2003 to 2007, it was noted that there was a 52% decrease in the hazard of death not preceded by relapse, and overall mortality was reduced by 41%.^10^ In our study, we have shown improved outcomes in patients who underwent allo-SCT in CR1, and we have also shown that RIC with Flu/Mel had significantly better outcomes compared with MAC with Bu/Cy in patients in CR1 allo-SCT.

In various studies that include a phase III randomized control trial comparing RIC with MAC, results showed no significant difference in nonrelapse mortality, incidence of relapse, disease-free survival, or OS.^23-25^ However, a prospective multicenter study conducted by CIBMTR (BMT-CTN 0901) was recently closed prematurely as a result of the high incidence of relapse in the RIC regimen arm.^26^ A major limitation of this study was that different RIC regimens were allowed in this study, and of the regimens used, a RIC regimen with busulfan was used in the majority, whereas a Flu/Mel regimen was used in < 20% of patients in this study. It has been previously reported that among RIC regimens, the Flu/Mel regimen had a significantly lower risk of relapse than other nonablative and RIC regimens.^27,28^ A recently reported direct comparison of the Flu/Mel regimen with a busulfan-based RIC regimen illustrated a significantly lower risk of relapse with the Flu/Mel regimen, and the overall outcomes with the Flu/Mel regimen in this study were similar to what we report here.^29^

In this study, we have demonstrated a significant long-term survival advantage with a well-tolerated Flu/Mel RIC regimen. The 5-year OS and EFS were significantly higher in the Flu/Mel arm compared with the Bu/Cy arm, even though the median age in the former group was one decade older. This can be attributed to lower TRM at both 100 days and 1 year in the Flu/Mel arm. This reduction in morbidity and mortality related to the conditioning regimen also contributes to reducing the medical cost of allo-SCT for patients with AML in CR1. In a smaller number of high-risk patients, as we defined it in this manuscript, we used a reduced toxicity MAC regimen with Flu/Bu to address the potential increased risk of relapse in this AML CR1 subset after introduction of RIC regimen for AML CR1 at our center. We found that results with the use of reduced toxicity Flu/Bu MAC regimen were comparable to those with the RIC Flu/Mel conditioning regimen; however, this analysis was limited by the small number of cases in the Flu/Bu arm (data not shown). On the basis of the exclusion of this high-risk subset in the Flu/Mel arm, this analysis does not allow us to conclude on the superiority of the Flu/Mel regimen over the other regimens, but it does demonstrate its favorable profile within the economic constraints in which we operate, and, in this context, it is likely more cost-effective than more intensive conditioning regimens for allo-SCT in AML CR1. A prospective and detailed cost analysis is required to address this in detail.

Finally, for a majority of our patients in India and those coming to our center, the medical expenses are self-paid, as we reported previously.^12^ It is our experience that the financial constraints for most patients are such that they have only one attempt at achieving cure and, as a result, subsequent salvage chemotherapy and allo-SCT in CR2 may not be an option in the majority. Compared with previously reported data from our center, the long-term outcome of patients receiving an RIC regimen, allo-SCT in CR1 as reported in this work, is significantly superior that for patients receiving chemotherapy alone, with an almost two-fold increase in long-term survival.^12^

A major limitation of this retrospective analysis is the absence of data on the total number of patients with AML who were diagnosed during the study period, the number who were eligible for allo-SCT, the number who actually underwent allo-SCT, and the reasons for not undergoing allo-SCT if they did not. Extrapolating from our recently reported prospective analysis over a relatively short period of time, we estimate that, of the young adult patients who opt for treatment, only 20% of patients who were eligible for allo-SCT actually underwent allo-SCT. The major reasons for not undergoing allo-SCT were lack of HLA matched related donors followed by lack of financial resources to proceed with it. In that analysis, there was an induction mortality of 25% in young adults, and the remaining patients received chemotherapy alone consolidation postinduction.^12^

In conclusion, considering the younger age of our patients, the improved long-term clinical outcomes with an Flu/Mel RIC regimen, and the setting of a self-paid-for medical care delivery system—as a result of which the majority of patients cannot afford salvage therapy in the event of a relapse—we feel these data would suggest that allo-SCT in CR1 using an Flu/Mel RIC regimen is likely the most cost-effective option for our patients. It is possible that this approach would likely be applicable to most developing countries with similar constraints and health care delivery systems.
